# Supplementary material for: Impact Assessment of Pituitary Adenylate Cyclase Activating Polypeptide (PACAP) and Hemostatic Sponge on Vascular Anastomosis Regeneration in Rats
Source: Int J Mol Sci. 2023 Nov 24;24(23):16695. doi: 10.3390/ijms242316695 (PMC10706260; doi:10.3390/ijms242316695)
Supplement: Supplementary file 1 [file ijms-24-16695-s001.zip › ijms-2655154-supplementary.pdf]

International Journal of *Molecular Sciences*

MDPI

Supplementary Materials: Impact Assessment of Pituitary Adenylate Cyclase Activating Polypeptide (PACAP) and Hemostatic Sponge on Vascular Anastomosis Regeneration in Rats

Laszlo Adam Fazekas, Balazs Szabo, Vince Szegeczki, Csaba Filler, Adam Varga, Zoltan Attila Godo, Gabor Toth, Dora Reglodi, Tamas Juhasz and Norbert Nemeth

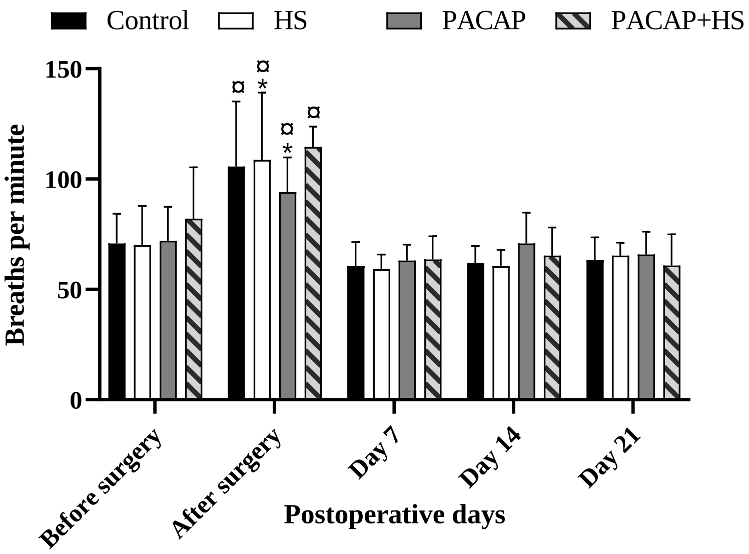

Figure S1. Breathing rate alterations in Control, HS, PACAP and PACAP+HS groups. Means±S.D., *p<0.05 vs. before surgery; ¤p<0.05 vs. Day 21

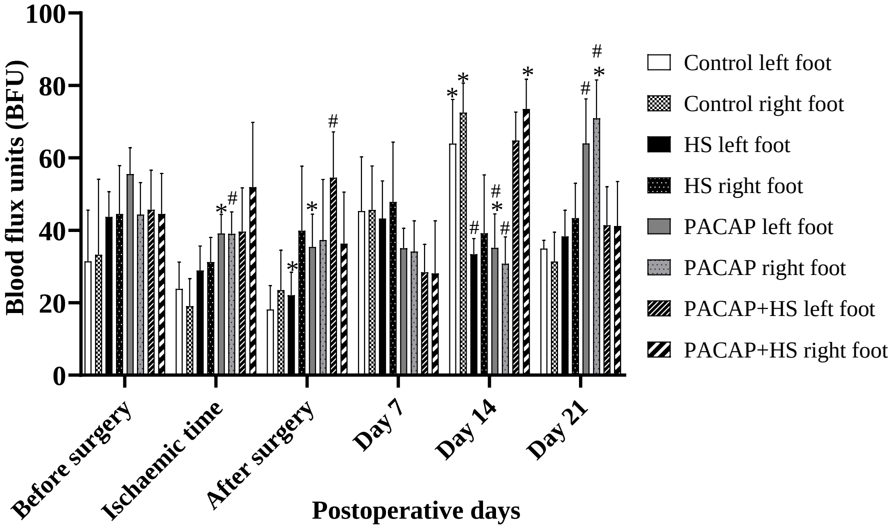

Figure S2. Changes of foot skin microcirculatory blood flux unit (BFU [au]) in Control, HS, PACAP and PACAP+HS groups. Means±S.D., *p<0.05 vs. before surgery; #p<0.05 vs. Control

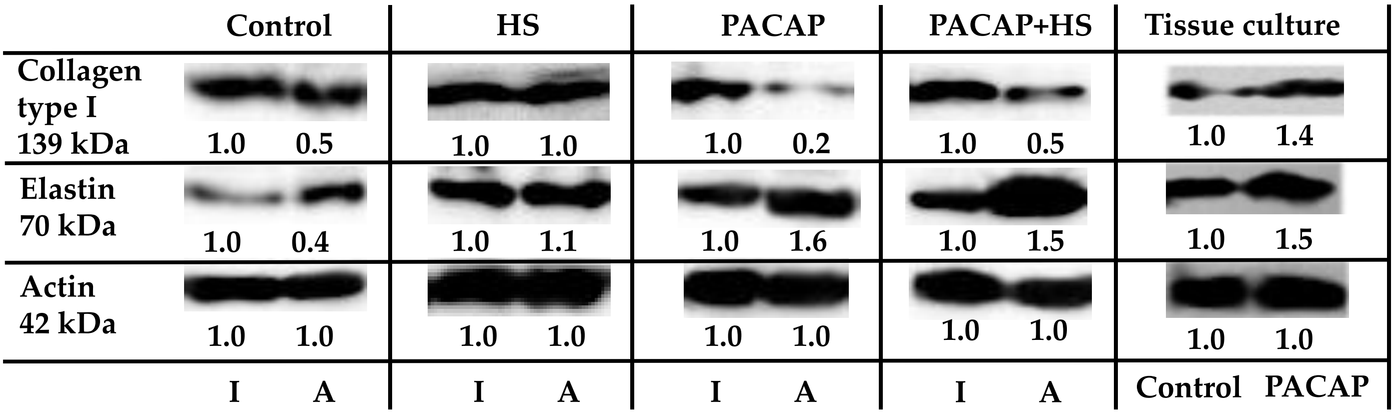

Figure S3. Comparative picture of representative western blot analysis on freshly excised arteries and tissue cultured arteries for collagen type I, elastin and actin in the experimental groups. I: intact, A: anastomosed

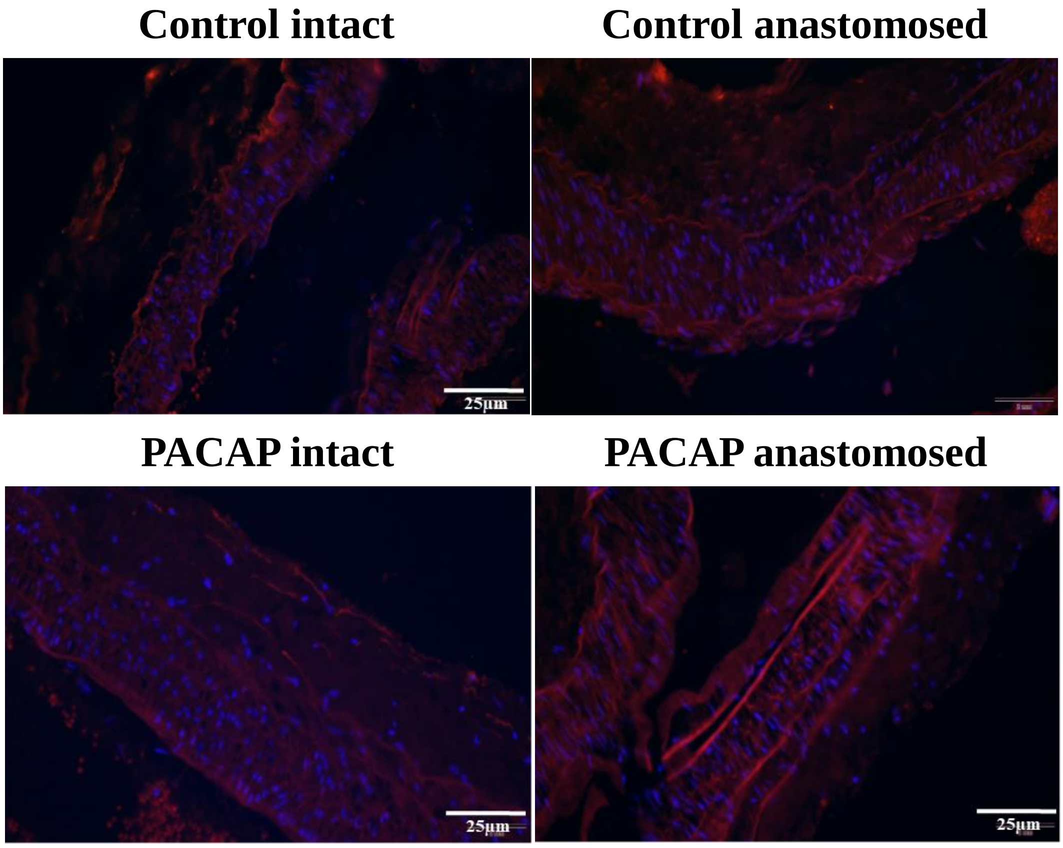

Figure S4. Representative pictures for elastin immunohistochemistry (original magnification: 40×) on tissue cultured arteries.

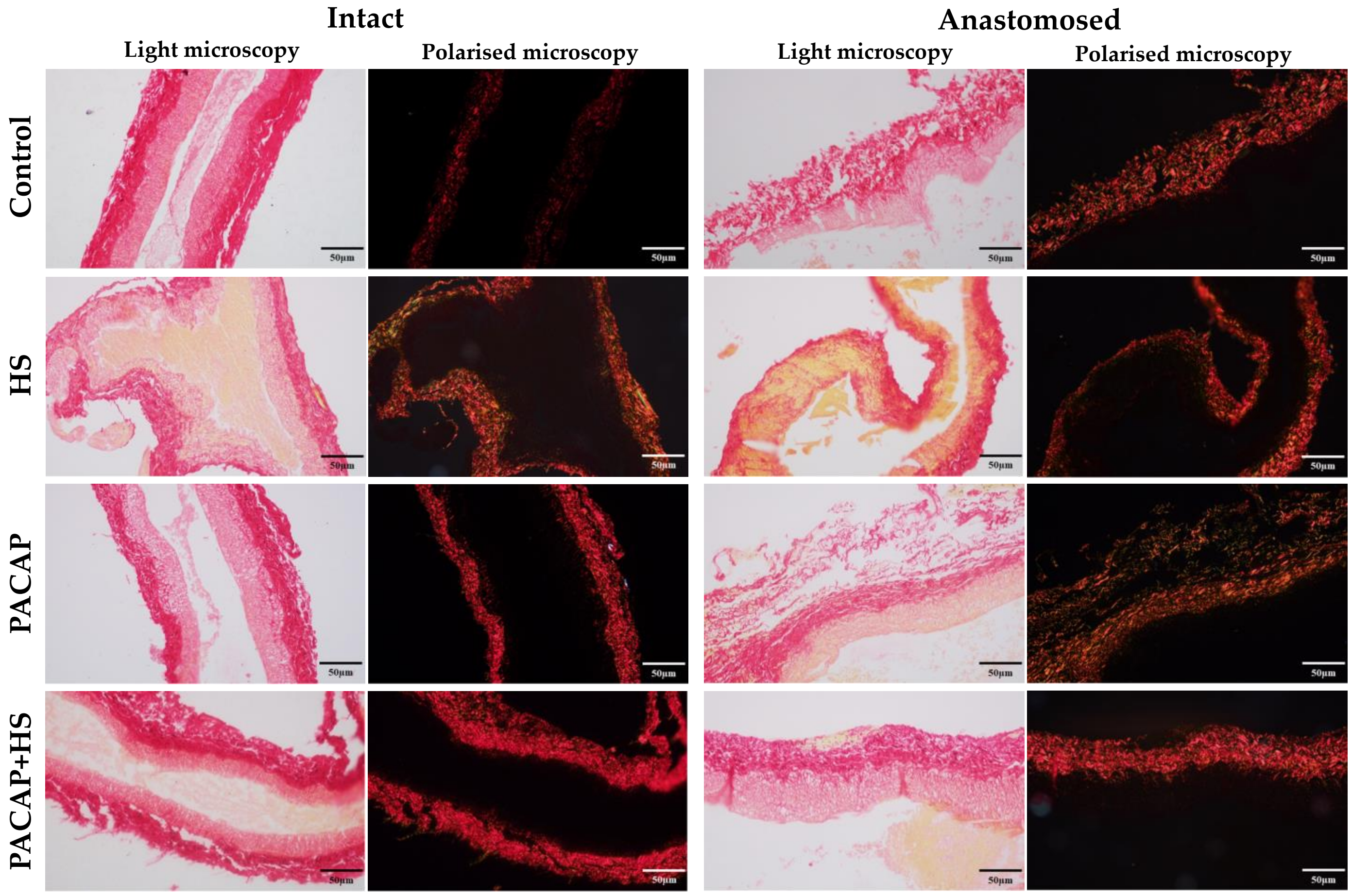

Figure S5. Representative picrosirius stained slides (original magnification: 20×) on freshly excised arteries in the experimental groups.

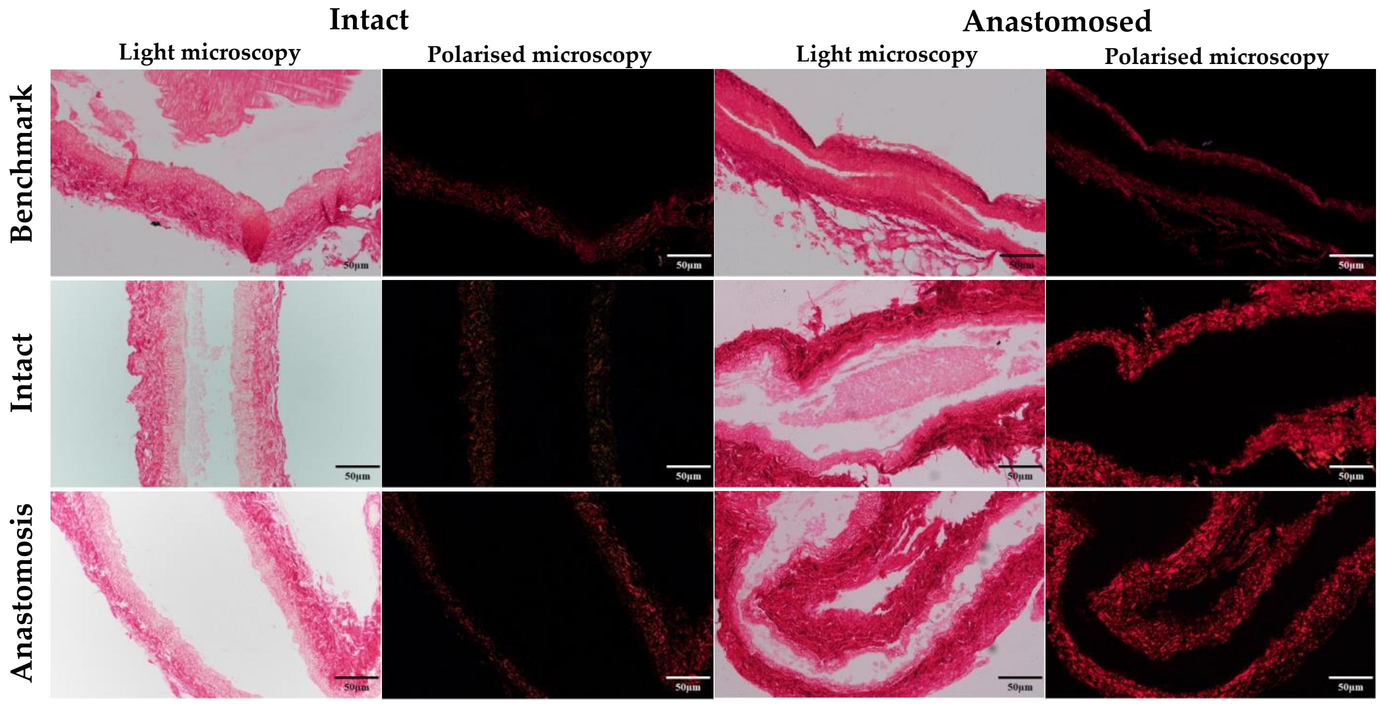

Figure S6. Representative picrosirius stained slides (original magnification: 20×) on tissue cultured arteries.

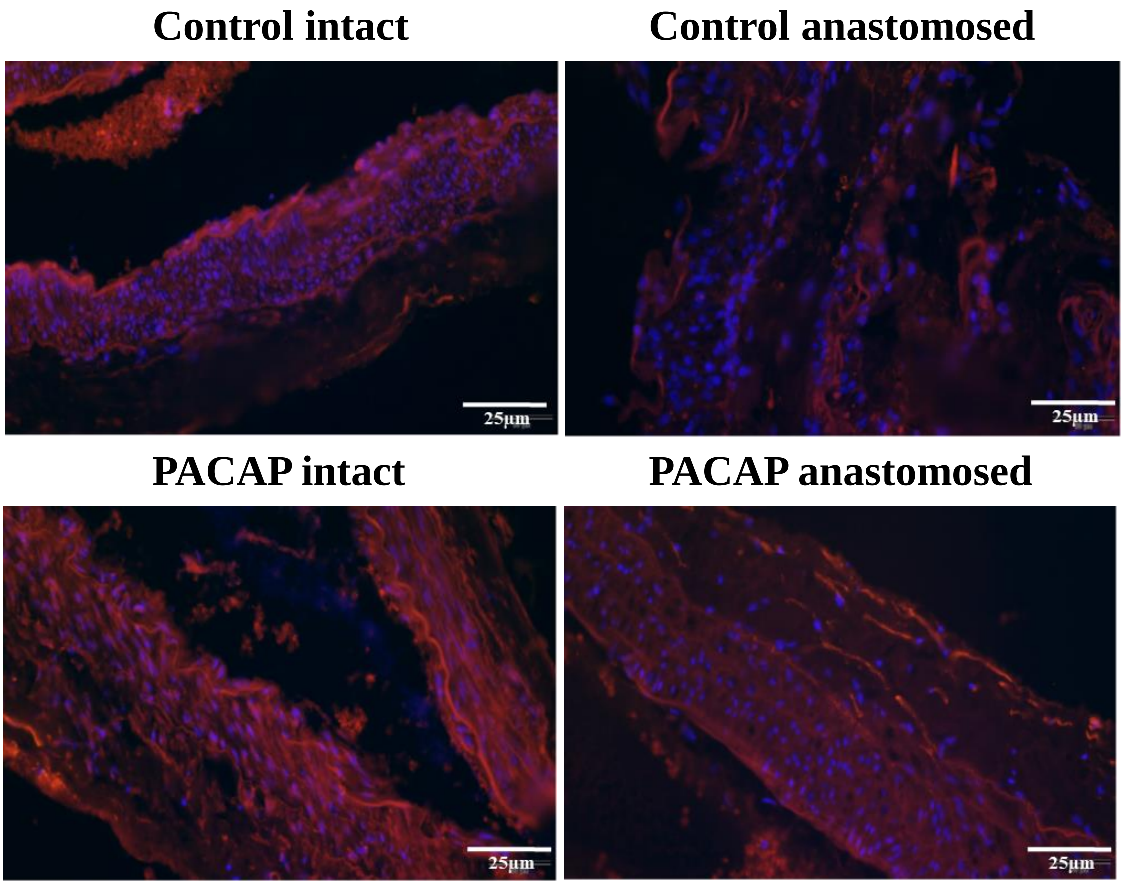

Figure S7. Representative pictures for collagen type I immunohistochemistry (original magnification: 40×) on tissue cultured arteries.
